# Isolated renal glucosuria due to *SLC5A2* gene mutation: a rare presentation

**DOI:** 10.1590/2175-8239-JBN-2024-0193en

**Published:** 2025-01-20

**Authors:** Priyanka Dua, Ashok Singh, Om P. Mishra

**Affiliations:** 1 Heritage Institute of Medical Sciences, Department of Pediatrics, Varanasi, India.

Dear Editor,

Isolated renal glucosuria (RG) is a rare genetic disorder marked by glucosuria without any other tubular abnormalities. It is characterized by the presence of glucose in urine with normal blood glucose levels in the absence of any other systemic and kidney diseases^1^.

A four-year-old female child presented with the symptoms of increased thirst and the complaint of accumulation of ants at the spot where she used to urinate as informed by her parents. There is no history of polyuria, weakness, muscle cramps, rapid deep breathing, burning during urination or significant weight loss. There was no family history of similar complaints.

Clinical examination was unremarkable. The height, weight and blood pressure were 105 cm, 14.3 kg and 92/56 mm Hg, respectively. Investigations revealed a hemoglobin of 11.3 g/dL, a total leucocyte counts of 9600/mm^3^, polymorphs 27%, lymphocytes 60%, eosinophils 3%, a platelet count of 2,64,000/mm^3^ and ESR of 14 mm/hr. Fasting blood glucose, post-prandial blood glucose, serum urea, creatinine, sodium and potassium, blood pH and HCO3^-^ levels were 82.0 mg/dL 87.0 mg/dL 29.6 mg/dL, 0.6 mg/dL 142.3 mEq/L, 4. 2 mEq/L, 7.36 and 20.4 mEq/L respectively. Urine analysis consistently showed presence of glucose + (100 mg/dL), with no proteinuria, hematuria or pyuria.

Clinical presentation failed to provide any definite diagnosis. Therefore, whole exome sequencing was performed, which revealed a SLC5A2 gene mutation, compound heterozygous likely pathogenic variant on intron 7 (c.885+5G>A, 5’ splice site) consistent with the diagnosis of renal glucosuria, and a variant of unknown significance on exon 12 (c.1616T > G, p. Leu539Arg) (Figure 1). We could not perform gene sequencing on the parents for segregation analysis due to financial constraints.

**Figure 1:**
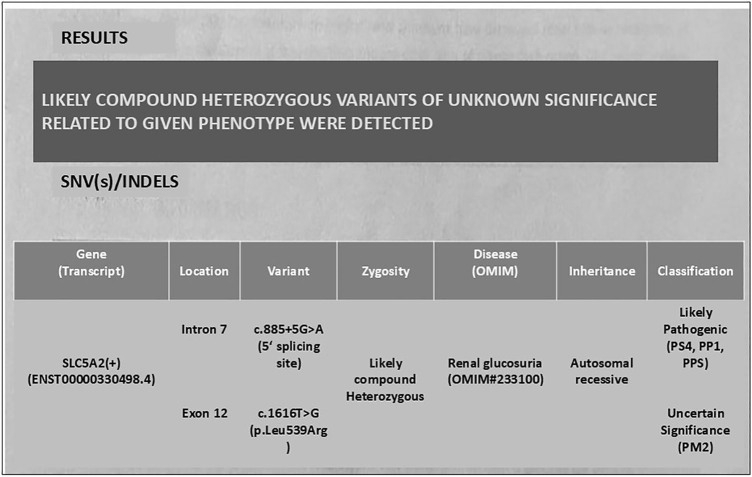
Result of genetic analysis.

## Discussion

RG is a condition that occurs due to variation in the SLC5A2 gene, responsible for the production of sodium-glucose cotransporter-2 (SGLT-2) which is the primary mediator of glucose reabsorption. Alterations in the SLC5A2 gene influence the activity of SGLT-2, which ultimately results in isolated glycosuria^1^. A total of 86 mutations in SLC5A2 gene including the present variant have been reported so far^2^. RG is considered an asymptomatic condition, with glucosuria being the only manifestation. Additionally, individuals with RG do not experience any clinical complications. RG is essentially considered a benign condition and usually no specific treatment is needed^3^. However, some cases of RG may present with episodic dehydration, polyuria, mild growth delays, and an increased risk for urinary tract infections^2^. Therefore, accurate diagnosis through genetic testing is vital to differentiate RG from other proximal tubular disorders and thus avoid parental anxiety and unnecessary diagnostic testing or intervention.
