# Supplementary material for: Isolated renal glucosuria due to SLC5A2 gene mutation: a rare presentation
Source: J Bras Nefrol. 2025 Jan 20;47(1):e20240193. doi: 10.1590/2175-8239-JBN-2024-0193en (PMC11772041; doi:10.1590/2175-8239-JBN-2024-0193en)
Supplement: Translation

# Translation: Glicosúria renal isolada devido à mutação no gene SLC5A2: uma apresentação rara

**DOI:** 10.1590/2175-8239-JBN-2024-0193pt

**Authors:** Priyanka Dua, Ashok Singh, Om P. Mishra

**Affiliations:** 1 Heritage Institute of Medical Sciences, Departamento de Pediatria, Varanasi, Índia.

Prezado Editor,

A glicosúria renal (GR) isolada é um distúrbio genético raro marcado pela presença de glicosúria sem quaisquer outras anormalidades tubulares. Caracteriza-se pela presença de glicose na urina com níveis normais de glicemia, na ausência de outras doenças sistêmicas e renais^1^.

Uma criança de quatro anos de idade, do sexo feminino, apresentou-se com sintomas de aumento da sede e, conforme relato de seus pais, com queixa de acúmulo de formigas no local onde costumava urinar. Não havia histórico de poliúria, fraqueza, cãibras musculares, respiração rápida e profunda, sensação de ardor ao urinar ou perda significativa de peso. Não havia histórico familiar de queixas semelhantes.

O exame clínico não apresentou alterações significativas. A altura, o peso e a pressão arterial da paciente foram de 105 cm, 14,3 kg e 92/56 mm Hg, respectivamente. Os exames revelaram hemoglobina de 11,3 g/dL, contagem total de leucócitos de 9600/mm^3^, polimorfonucleares 27%, linfócitos 60%, eosinófilos 3%, contagem de plaquetas de 2.64.000/mm^3^ e VHS de 14 mm/h. Os níveis de glicemia de jejum, glicemia pós-prandial, ureia, creatinina, sódio e potássio séricos, pH sanguíneo e HCO3*-* foram de 82,0 mg/dL, 87,0 mg/dL, 29,6 mg/dL, 0,6 mg/dL, 142,3 mEq/L, 4,2 mEq/L, 7,36 e 20,4 mEq/L, respectivamente. A urinálise mostrou consistentemente a presença de glicose + (100 mg/dL), sem proteinúria, hematúria ou piúria.

A apresentação clínica não permitiu estabelecer um diagnóstico definitivo. Portanto, foi realizado o sequenciamento completo do exoma, que revelou uma mutação no gene SLC5A2, uma variante heterozigota composta provavelmente patogênica no intron 7 (c.885 + 5G > A, sítio de splicing 5’), consistente com o diagnóstico de glicosúria renal, e uma variante de significado desconhecido no exon 12 (c.1616T>G, p. Leu539Arg) (Figura 1). Não foi possível realizar o sequenciamento genético nos pais para análise de segregação devido a restrições financeiras.

**Figura 1:** Resultado da análise genética.

## Discussão

A GR é uma condição que ocorre devido à variação no gene SLC5A2, responsável pela produção do cotransportador de sódio-glicose-2 (SGLT-2), que é o principal mediador da reabsorção de glicose. As alterações no gene SLC5A2 influenciam a atividade do SGLT-2, resultando, em última análise, em glicosúria isolada^1^. Um total de 86 mutações no gene SLC5A2, incluindo a presente variante, foram relatadas até o momento^2^. A GR é considerada uma condição assintomática, sendo a glicosúria a única manifestação. Além disso, indivíduos com GR não apresentam complicações clínicas. A GR é essencialmente considerada uma condição benigna e geralmente não requer tratamento específico^3^. No entanto, alguns casos de GR podem apresentar episódios de desidratação, poliúria, atrasos leves no crescimento e um risco aumentado de infecções do trato urinário^2^. Portanto, o diagnóstico preciso por meio de testes genéticos é fundamental para diferenciar a GR de outros distúrbios do túbulo proximal e, assim, evitar a ansiedade dos pais e a realização de testes ou intervenções diagnósticas desnecessárias.
